# Application of Baltic Pine (*Pinus sylvestris*) Needle Extract as a Gut Microbiota-Modulating Feed Supplement for Domestic Chickens (*Gallus gallus*)

**DOI:** 10.3390/plants12020297

**Published:** 2023-01-08

**Authors:** Juris Rubens, Juris Kibilds, Martins Jansons, Inga Piginka-Vjaceslavova, Ilze Barene, Irena Daberte, Laima Liepa, Aija Malniece, Arturs Rubens, Vytaute Starkute, Egle Zokaityte, Modestas Ruzauskas, Elena Bartkiene, Vadims Bartkevics, Iveta Pugajeva

**Affiliations:** 1 Research and Experimental Development on Biotechnology, BF-ESSE LLC, Brivibas Gatve 369 k-2, LV-1024 Riga, Latvia; 2 Institute of Food Safety, Animal Health and Environment “BIOR”, Lejupes 3, LV-1076 Riga, Latvia; 3 Department of Applied Pharmacy, Faculty of Pharmacy, Riga Stradins University, Dzirciema 16, LV-1007 Riga, Latvia; 4 Faculty of Veterinary Medicine, Latvia University of Life Sciences and Technologies, Kristapa Helmana 8, LV-3004 Jelgava, Latvia; 5 Institute of Animal Rearing Technologies, Faculty of Animal Sciences, Lithuanian University of Health Sciences, Mickeviciaus 9, LT-44307 Kaunas, Lithuania; 6 Institute of Microbiology and Virology, Faculty of Veterinary, Lithuanian University of Health Sciences, Mickeviciaus Str. 9, LT-44307 Kaunas, Lithuania; 7 Faculty of Chemistry, University of Latvia, Raina Blv 19, LV-1586 Riga, Latvia

**Keywords:** gut microbiota, antimicrobial activity, feed supplements, phytobiotics, Scots pine

## Abstract

The valorization of wood industry residues is very desirable from a circular economy perspective. Pine needle extracts are known for their health-promoting properties and therefore can be used as herbal remedies and nutritional supplements. Since the withdrawal of antibiotics as growth promoters in the European Union, natural feed additives that improve poultry health and production are needed. It was proposed that pine needle extract could be a good alternative to antibiotic usage at sub-therapeutic concentrations. The results relevant to our assumption could be obtained by using domestic chickens as an in vivo model for the evaluation of gut microbiota-altering properties of pine needle extract as an herbal supplement. We tested the antimicrobial effects of Baltic pine (*Pinus sylvestris*) needle extract. Then, we used chicken (*Gallus gallus*) that received feed supplemented with two different concentrations of the extract for 40 days to evaluate the changes in gut microbiota using 16S rRNA gene sequencing. This preliminary study demonstrated trends toward dose-dependent desirable changes in broiler microbiome, such as a reduction in the relative abundance of *Campylobacter*.

## 1. Introduction

The forestry sector is an important part of the economy in many countries. Pine residues and by-products contain compounds of high industrial interest, while the demand for herbal remedies, nutritional supplements, and functional foods has been increasing worldwide [1]. Pine extracts have attracted the attention of researchers because of their purported health benefits [2]. Several growth- and health-promoting properties have been attributed to certain plant-derived products that may be further exploited in the poultry industry. These benefits are derived by improving gut health, including increasing the digestibility of feed, modifying digestive secretions, as well as sustaining and improving the gut structure. Furthermore, some phytobiotics stabilize the microbiome, thus reducing the production of microbial toxins [3]. The positive effect of phytobiotics is mainly linked to active plant constituents, including terpenoids (mono- and sesquiterpenes, steroids), phenolics (tannins), glycosides, alkaloids that may be present as conjugates, flavonoids, and glucosinolate [4]. Various parts of woody herbs (needles, shoots, etc.) in the form of infusions, extracts, and ointments have been historically used in folk and traditional medicine. The phytochemical constituents present in conifer extracts are nontoxic at therapeutic levels, with polyphenolic compounds often exhibiting significant biological effects. Stilbenes, terpenes, alkaloids, lignins, and flavanoids, such as quercetin, rutin, resveratrol, and the compounds PYC and enzogenol, are the phytochemical components of conifer extracts reportedly having sedative, antidiabetic, anticancer, and anesthetic effects [5]. Pine needles, being one of the products derived from coniferous trees, have been used in traditional Chinese medicine to treat diseases, such as wind-cold-dampness arthralgia, traumatic injury, sleeplessness, eczema, and edema. The medicinal properties of pine needles are assumed to be related to their bioactive substances such as carotenoids, terpenoids, phenolic compounds, tannins, and alkaloids [6]. It has been reported that pine needles have shown anti-inflammatory and anti-bacterial activity against *E. coli, S. aureus*, and *B. subtilis* in vitro studies [7]. Another study indicated that aqueous extracts of pine needles possessed a spectrum of antioxidant and DNA-protective properties [8]. Hoai et al. indicated that *Pinus sylvestris* L. needle extract and essential oil exhibited some potential as a chemopreventive or chemotherapeutic agent for mammary tumors unresponsive to endocrine treatment [9].

Antibiotic usage has enhanced the health and well-being of poultry by reducing the incidence of disease and has facilitated efficient production of poultry products [10]. The risk concerning residues of antibiotics in edible tissue and products that can produce allergic or toxic reactions in consumers is known to be negligible because only antibiotics that are not absorbed in the digestive tract have been authorized as growth promoters [11]. However, concerns about the development of antimicrobial resistance and the transfer of antibiotic resistance genes from animal to human microbiota have led to the withdrawal of approval for antibiotics as growth promoters in the European Union since 1 January 2006 [12]. Therefore, natural feed additives that improve poultry health and production are needed.

The gut microbiota comprises microorganisms resident in the digestive tract of the host. The gut microbiota is closely linked with the health and disease status of the host. In recent years, a large number of studies have demonstrated that diet influences the composition of animal gut microbiota [13]. The microbiota of chickens are well differentiated across the gastrointestinal compartments (crop, proventriculus, gizzard, duodenum, ileum, cecum, and colon) due to different physicochemical conditions, mainly the pH, growth substrate availability, redox potential, and the antimicrobial activity of host secretions. Moving through the gastrointestinal tract, the availability of growth substrates decreases [14]. The crop, proventriculus, and gizzard are dominated by *Lactobacilli* due to the strong selection by pH. The microbiota is significantly diverse only in the cecum and colon [15]. Due to the decreasing redox potential from the proximal to the distal intestine, the proximal intestine also supports the growth of facultative anaerobic bacteria and the small intestines from healthy chickens contain few strict anaerobes. The cecum and colon are characterized by the presence of rich microbiota dominated by strict anaerobes, which are usually specialized in utilizing feed that is not digested by the host, e.g., resistant starch or proteins, or carbohydrate fractions excluding starch and free sugars (non-starch polysaccharides) [14]. The total count of microorganisms is low in the small intestine (approx. 10^5^ CFU per gram of digesta), and very high in the cecum (approx. 10^10^ CFU per gram of digesta, containing approximately 1000 species). In a healthy adult chicken, the cecum is usually colonized by Gram-positive *Firmicutes* and Gram-negative *Bacteroides*, which constitute approximately 90% of all microbiota, and the remaining phyla are usually Gram-positive *Actinobacteria* and Gram-negative *Proteobacteria*. Although this is the average composition of the microbiota in the chicken cecum, there can be highly individual variations without signs of abnormality. However, all these four phyla are always found in the ceca of normal adult chickens [15]. It was reported that the broiler chicken may be a useful model for initial in vivo screening of Fe bioavailability in foods due to its growth rate, anatomy, size, and low cost. Some researchers have even suggested that such a model could be useful as an intermediate source of in vivo observations in preparation for subsequent human studies [16].

Considering the scientific data about the anti-inflammatory and anti-bacterial effects of pine needles towards different pathogenic bacteria [7], it has been proposed that pine needle extract could be a good alternative to antibiotic usage at sub-therapeutic concentrations for enhancing the growth and health of poultry. The purpose of this pilot study was to evaluate the antimicrobial activity of the Baltic pine (*Pinus sylvestris*) needle extract against bacterial strains and to perform a pilot study using the domestic chicken (*Gallus gallus*) as an in vivo model to evaluate the properties of pine needle extract as a dietary supplement on the chicken gut microbiota under non-challenged conditions.

## 2. Results

### 2.1. The Antimicrobial Activity of Baltic Pine (Pinus sylvestris) Extract

The antimicrobial properties of Baltic pine (*Pinus sylvestris*) needle extract evaluated in liquid medium are shown in Table 1. It was established that 500 µL of pine needle extract was insufficient for inhibiting the growth of the tested pathogen in liquid medium; however, 1000 µL of pine needle extract did inhibit *Bacillus cereus* and 1500 µL of pine needle extract inhibited *Enterobacter cloacae*, *Bacillus cereus*, *Salmonella enterica*, and *Acinetobacter baumanii* strains.

**Figure 1:**
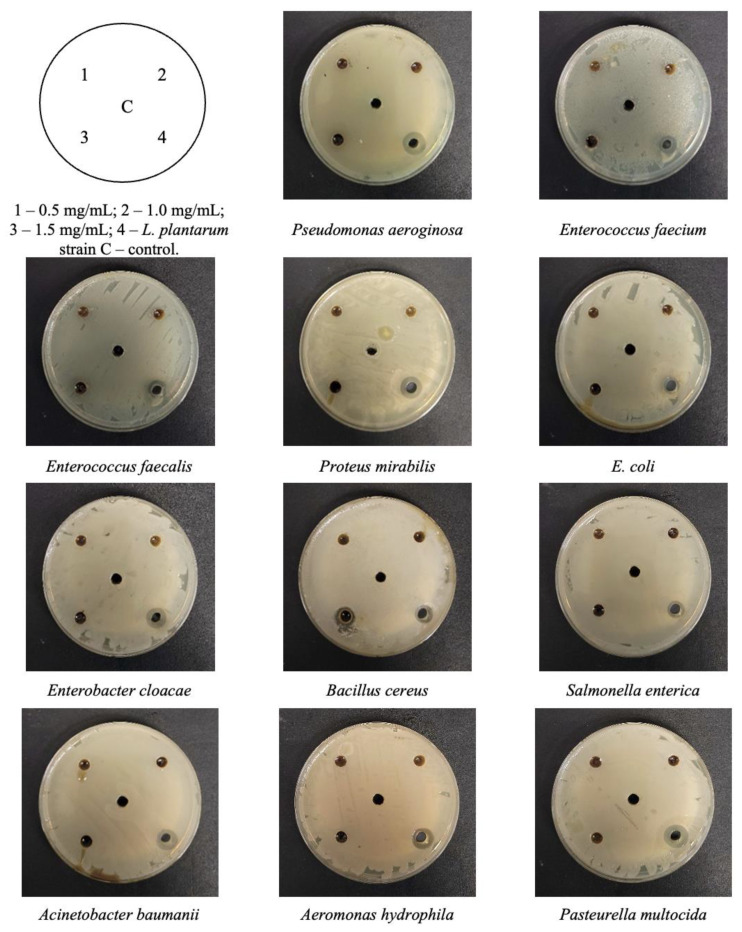
Images showing the diameters of inhibition zones (DIZ) by pine extract and *L. plantarum* strain against the tested pathogens.

The diameters of inhibition zones (DIZ, mm) for the pine extract and *L. plantarum* strain against pathogenic opportunistic strains are shown in the Figure 1 and Table 2.

### 2.2. Summary of 16S rRNA Gene Sequencing

In total, 6,423,182 paired-end reads were generated from the fifteen samples, ranging from 361,090 to 489,066 reads per sample. After the trimming and denoising steps, 2,144,509 features remained. The mean number of features per sample was 142,967, from the lowest coverage of 121,506 features to the highest of 158,486. Alpha diversity rarefaction curves were generated to verify that sufficient sequencing depth had been achieved. Saturation of Shannon diversity index and the observed feature richness was achieved at approximately 10,000 and 110,000 features, respectively, for all three groups assigned to the following diets. Examination of the reading counts and taxonomic profiles of the negative control and mock community did not raise any concerns of contamination or poor performance of the sequencing process.

### 2.3. Diversity of Cecal Microbiota and the Trends Observed in Feeding Groups

**Figure 2:**
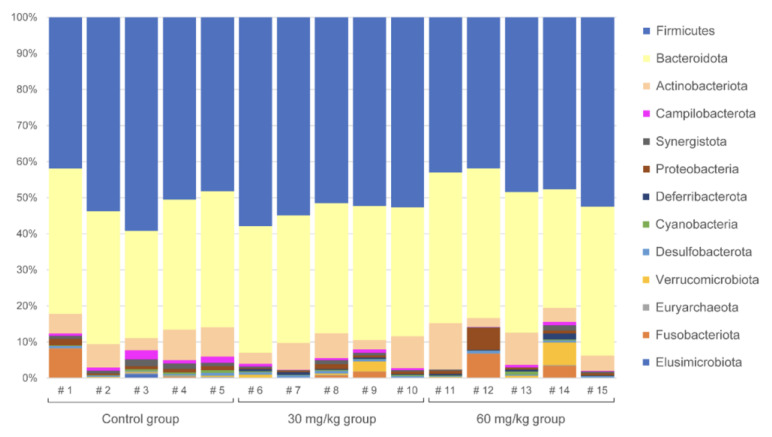
Phylum-level composition of the cecal microbiota in chicken. Three groups (five replicates in each group) represent the different treatments, as indicated.

The major bacterial phyla that were observed were *Firmicutes* (53.44% average relative abundance), *Bacteroidota* (33.14%), *Actinobacteriota* (6.43%), *Fusobacteriota* (1.60%), and *Proteobacteria* (1.37%). For the rest of phyla, the abundance was less than 1% on average. The composition of each sample at the phylum level is presented in Figure 2.

Next, the effect of pine needle extract as a feed supplement on the alpha diversity of cecal microbiomes was evaluated. Three different alpha diversity measures were calculated. The Spearman correlation revealed a significant relationship (*p* = 0.0014) between the observed feature count and denoised read count per sample, which was expected, as more spurious ASVs can appear with increased depth of sequencing. However, other alpha diversity indices did not correlate with the sequencing depth, thus it was assumed that the other diversity measures were not biased by sample read counts. The observed feature count and Simpson’s index did not significantly correlate with the dietary supplement concentration, whereas Shannon’s index showed negative correlation with the dietary supplement dose (ρ = −0.6236, *p* = 0.013).

In order to evaluate the between-sample (beta) diversity, Bray–Curtis and generalized UniFrac distances were calculated between all samples. There was no significant correlation between the beta diversity metrics and read count, thus assuring that these metrics were also not biased according to the sequencing depth. Both Bray–Curtis and generalized UniFrac beta diversity metrics showed a significant positive correlation with the feed supplement dose (ρ = 0.473644, *p* = 0.001 and ρ = 0.267187, *p* = 0.003, respectively).

Eleven differently abundant genera in the cecal microbiota from birds fed a pine needle extract-supplemented feed as compared to the control group were identified using a general linear model framework (Table 3). Four of them, belonging to the families of *Bacteroidaceae*, *Marinifilaceae*, *Prevotellaceae*, and *Deferribacteraceae*, were more abundant when the feed was supplemented with pine needle extract. Seven other genera were less abundant when the birds were fed with supplemented feed: two belonging to the family *Rikenellaceae* and the others belonging to *Barnesiellaceae*, *Campylobacteraceae*, *Methanobacteriaceae*, *Lachnospiraceae*, and *Synergistaceae*.

## 3. Discussion

The antimicrobial activity of pine needles has been widely studied and different bioactive compounds have been found to be responsible for its antimicrobial effects, e.g., terpenes, polyphenols, stilbenes, and tannins. The bioactive compounds of pine needles act as antimicrobials because they degrade microbial cell walls. The disruption of the cytoplasmic membrane and membrane proteins, cell leakage, cytoplasm coagulation, and proton motive force depletion are all examples of their inhibitory action [5].

In our study, the antimicrobial effects of pine needle extract were proven for *Enterobacter cloacae*, *Bacillus cereus*, *Salmonella enterica*, and *Acinetobacter baumanii* strains by using the agar well diffusion assay. It has been reported that *Enterobacter cloacae* causes wound, respiratory, and urinary tract infections, possibly leading to bacteremia in the case of strains producing extended-spectrum β-lactamase [17]. *Bacillus cereus* has been associated with severe infections in immunocompromised hosts and can cause *septicemia* as well as *endophthalmitis*, which can lead to vision loss [18]. *Salmonella enterica* represents the most widespread pathogenic species and includes >2600 serovars characterized thus far [19]. *A. baumannii* causes a range of infections in both the hospital and community, including skin and soft tissue infections, urinary tract infections, meningitis, bacteremia, and pneumonia, with the latter being the most frequently reported infection in both settings [20]. However, further studies are needed to indicate the minimal effective concentration of Baltic pine (*Pinus sylvestris*) needle extract against a variety of pathogenic strains.

Other studies have shown that the combinations of different ingredients enhance the antimicrobial activity and inhibit a broader spectrum of pathogenic bacteria, compared to isolated ingredients and might promote the effectiveness of the antimicrobial product by allowing a reduction in the overall dose [21]. In this regard, we propose that a combination of Baltic pine (*Pinus sylvestris*) extract with LAB bacteria may be promising [22]. In addition, the synergism of antimicrobials could be applied in an attempt to prevent or delay the emergence of resistant populations of pathogenic organisms in vivo [20].

The microbial community present in the gastrointestinal tract has been widely associated with different factors affecting the health of chickens, such as the immune system, the physiology of the digestive system, and the suppression of pathogens, as well as the performance in production. Feed additives are widely used to improve chicken gut health and to stimulate performance [13,23]. According to the bacterial meta-analysis of chicken cecal microbiota, *Firmicutes* was the most prevalent phylum, followed by *Bacteroidetes* and *Proteobacteria* [24]. The prevalence of *Firmicutes* and *Bacteroidetes* have been associated with their capacity for digesting cellulose and non-starch polysaccharides, that cannot be digested in the small intestine and leads to short-chain fatty acid production [25]. In our study, a similar abundance profile was observed for *Firmicutes* and *Bacteroidetes*, but the next most abundant phylum was *Actinobacteriota*, followed by *Fusobacteriota* and then *Proteobacteria*. However, the distribution of *Fusobacteriota* was very uneven among the chickens tested in our study.

It was observed that the Shannon’s alpha diversity index significantly correlated with the dose of dietary supplement while the Simpson’s index did not, indicating that the shift in microbiome composition mainly affected the minority taxa, not the dominant ones, since the Shannon’s index gives more weight to species richness and thus is more sensitive towards minor features [26]. The significant correlation between the beta diversity measures and the difference in the feeding supplement dose received by each chicken demonstrated that a general shift in the cecal microbiome composition was observed despite the small sample size and notable inter-individual variation in microbiome profiles (see Figure 1).

Among the four taxa that were more abundant when the feed was supplemented with pine needle extract were a Gram-negative genus of *Bacteroides* that are common gut microbiota members in all endothermic animals [27]. *Bacteroides* play an important role in breaking down complex macromolecules and generate acetate and propionate as major fermentation products [28]. *Odoribacter* is capable of butyrate production via lysine fermentation and succinate reduction [15]. Butyrate, a short-chain fatty acid that directly stimulates an increase in the absorptive surface area, suppresses the growth of zoonotic pathogens, induces the expression of host-defense peptides, and modulates host epigenetic regulation [29]. Chicken isolates belonging to the family *Prevotellaceae* have not yet been characterized in detail. In vitro culturomic studies have indicated that chicken *Prevotellaceae* are specialized in the digestion of complex polysaccharides and dominate in the microbiota when feed enriched in vegetable fiber is common [15]. Among the minor phyla associated with the mature microbiota, there was a noteworthy presence of bacteria with the potential to stimulate mucus layer formation, which are therefore associated with a healthy gut, like *Mucispirillum* found only in free-range, slow-growing chickens and at higher levels in 81-day old, free-range, slow-growing chickens where it has been recognized as a biomarker [30].

For a few bacterial taxa belonging to the families *Barnesiellaceae* and *Rikenellaceae*, decreased relative abundance was observed when the birds were fed with supplemented feed. These taxa belong to the order *Bacteroidales* that encompass Gram-negative anaerobic coccobacilli, with saccharolytic and proteolytic activities. *Barnesiellaceae* is a proposed taxonomic group that has not yet been characterized. *Rikenellaceae* have been found enriched in the ceca of mice with high-fat diet-induced obesity and seem to be highly susceptible to perturbations in the gut microbiota, such as those caused by antibiotics or probiotics supplementation [28].

The same decrease was observed for *Methanobrevibacter*. Many methanogenic archaea, or methanogens, use H_2_ and CO_2_ as substrates to synthesize methane. As the only producers of enteric methane, methanogens are responsible for the contribution of livestock industries to climate change and have thus become the focus of research toward developing mitigation strategies. The sequences of 16S rRNA genes closely related to certain species belonging to the genus *Methanobrevibacter* are among the most frequently found sequences in gastrointestinal tract samples from livestock [31].

One of the main functions of the cecum is bacterial fermentation of indigestible polysaccharides to produce short-chain fatty acids that can be absorbed by the host’s epithelial cells. This role is fulfilled by certain classes of *Firmicutes* (such as *Lachnospiraceae* or *Ruminococcaceae*) and *Bacteroidetes* [32]. The bacteria of the *Lachnospiraceae* family are absent or very sparse in young chickens [23]. There are phyla and genera that may appear in the microbiota of adult hens but are not universally distributed in all individuals, and these include Synergistetes (*Cloacibacillus* sp.) [15].

*Campylobacteriosis* is the most common bacterial gastroenteritis caused by *Campylobacter spp*. bacteria. A previous study in Latvia that had focused on the prevalence of *Campylobacter* in broiler production revealed an occurrence rate of 50.6% for *Campylobacter* [33]. Our preliminary study on chickens reared on an organic farm also indicated a high prevalence of *Campylobacter* spp. bacteria, indicating an urgent need for well-balanced nutrition in poultry farms where antibiotics cannot be used and highlights the problems faced by organic farmers. Furthermore, the reduction of *Campylobacter* loads or even eradication of certain *Campylobacter* species from the chicken gut would entail a benefit for public health. Notably, the relative abundance of *Campylobacter* in animals receiving 30 mg of pine needle extract per kg of b.w. was still similar to control samples. The abundance of said bacteria decreased significantly only with a higher dose (60 mg/kg), indicating that the lower dose would not be sufficient to achieve the desired effect on the cecal microbiome. This observation regarding the reduction of pathogenic bacteria should be investigated in further studies.

## 4. Materials and Methods

### 4.1. Baltic Pine (Pinus sylvestris) Needle Extract

Pine needle extract was obtained from the green coniferous biomass of Baltic pines (*Pinus sylvestris*). *Pinus sylvestris* have been given the status of Novel Food by the European Food Safety Agency [34]. Consequently, this valuable plant biomass can be converted into several types of high value-added products such as herbal remedies, nutritional supplements, and functional foods.

Pine needle extract was prepared by a biorefinery process using an organic solvent for the extraction of green wood conifers, followed by the separation of coniferous wax, treatment with aqueous alkaline solutions, acidification with mineral and organic acids, settling, layer-by-layer separation, and removal of solvent traces by distillation [35]. This industrial method makes it possible to obtain the most complete conifer needle extract containing more than one hundred hydrophobic and hydrophilic biologically active compounds in natural ratios. The obtained natural product in the form of a dark green mass with the characteristic smell and taste of pine is also known as chlorophyll–carotene paste. The main chemical components of pine needle extract are sodium chlorophyllin and other chlorophyll derivatives (4–16 g/L), β-carotene and other carotenoids (200–1200 mg/L), vitamin E (300–500 mg/L), vitamin K group (12–20 mg/L), sodium salts of fatty, resin dibasic, oxo-, and oxyacids (44–60%), minerals (5–7%), waxes (5–8%), phytosterols, polyphenols, and squalene [36].

### 4.2. Evaluation of the Antimicrobial Activity of the Pine Needle Extract against Bacterial Strains

The antimicrobial activity of Baltic pine (*Pinus sylvestris*) needle extract was assessed against 15 bacterial strains that were previously isolated from clinical material from animals (*Pseudomonas aeruginosa*; *Enterococcus faecium*; *Enterococcus faecalis*; *Proteus mirabilis*; *Escherichia coli*; *Klebsiella pneumoniae*; *Staphylococcus aureus*; *S. haemolyticus*; *Enterobacter cloacae*; *Citrobacter freundii*; *Bacillus cereus*; *Salmonella enterica*; *Acinetobacter baumanii*; *Aeromonas hydrophila*; *Pasteurella multocida*). The agar diffusion well assay and antimicrobial activity in liquid medium were assessed.

The antimicrobial activity of the extract in the liquid medium was evaluated according to the method described by Bartkiene et al. [37]: the extract was diluted 1:10 (*w*/*w*) with sunflower oil. Then, to the different amounts of diluted extract (500, 1000, and 1500 µL), 10 µL of 0.5 McFarland standard density bacterial cultures were added, mixed, and incubated at 35 °C for 24 h. After incubation, the viability of bacterial strains in the pine extract was assessed by plating them on a universal solid medium—Tryptone Soya Agar (Oxoid, Basingstoke, UK), followed by incubation at 35 °C for 48 h. The results were interpreted as (−) if the pathogens did not grow on the medium and (+) if the pathogens grew on the medium. The experiments were performed in triplicate.

For the agar well diffusion assay, suspensions of 0.5 McFarland standard of each bacterial strain were inoculated onto the surface of cooled Mueller–Hinton agar (Oxoid, Basingstoke, UK) using sterile cotton swabs. Wells with a 6 mm diameter were punched into the agar and filled with 50 µL of the extract (the concentrations tested were 0.5, 1.0, and 1.5 mg/mL; the dilutions were performed with sunflower oil). In addition, the antimicrobial properties of the pine needle extract were compared to the antimicrobial activity of the *Lactobacillus plantarum* strain using the same pathogens. *L. plantarum* was cultured in the MRS medium (Biolife, Italy) at 30 °C. Two percent of the MRS solution (*v*/*v*) in which the strain was cultured was inoculated into fresh medium and propagated for 18 h (viable LAB count 8.9 × 10^10^ CFU/mL). Wells with a 6 mm diameter were punched into the agar and filled with 50 µL of the L. plantarum culture in the MRS solution. The antimicrobial activity against the tested pathogens was established by measuring the diameters of the inhibition zones (DIZ). The experiments were repeated three times and the average DIZ (mm) was calculated.

### 4.3. Experimental and Sampling Procedures for the In Vivo Model

A total of 60 healthy 21-day old domestic chickens (*Gallus gallus*) of different crossbreeds grown for commercial purposes were randomly divided into three groups with twenty replicates. The feed of each group was supplemented with different amounts of pine needle extract. Chickens were reared on an organic farm where the birds were kept separately indoors and outdoors in specially designed aviaries. Feed and water were offered *ad libitum*. The three groups were on the following diets: group A received the standard non-supplemented diet; group B was fed a standard diet supplemented with pine needle extract at the concentration of 30 mg of extract per kg of bird body weight (b.w.); and group C was fed a standard diet supplemented with pine needle extract at the concentration of 60 mg extract per kg of b.w. The supplemented feed was prepared once a week, taking into account the actual weight of birds, replicates, daily feed intake, and the time period (Table 4). No differences were observed between the chicken groups regarding the willingness to consume non-supplemented feed and feed supplemented with pine needle extract.

Before the experiment, we confirmed that the composition of needle extract did not change during long-term storage, and the absence of microbial contamination was verified. Previous preliminary studies (data not published) showed that the optimal dose for consumption was assumed to be 30–60 mg pine needle extract per kg of animal weight. Based on this data, the amount of supplement to added to the feed was calculated.

The commercially available feed that is intended for feeding laying hens from the first day of life to 8 weeks of age was used as a basal diet and consisted of wheat, oat, soybeans, *Saccharomyces cerevisiae*, calcium carbonate, vitamin A, vitamin D_3_, vitamin E, vitamins B, vitamin K_3_, and trace elements: iron, zinc, manganese, copper, cobalt, selenium, as well as digestion promoters. The chemical composition of the feed consisted of: metabolizable energy (ME) 13.4 MJ/kg, crude protein 21.5%, crude fiber 4.54 %, calcium 1.0%, phosphorus 0.72 %, sodium 0.17%, lysine 1.05%, and methionine 0.30%.

The duration of the experiment was 40 days, which was adjusted to the usual life cycle of commercial poultry production, after which five individuals per treatment were randomly selected and slaughtered via cervical dislocation using the usual slaughtering procedure at the farm. The cecal contents were collected in a special sterile box for microbiome analysis. The samples were sent to the laboratory while packed in ice.

The management of birds was implemented by strictly following the recommendations of the Food and Veterinary Service of the Republic of Latvia. The birds were reared on an organic farm and slaughtered at the farm according to the standard process for meat production, in line with the requirements of the Council Regulation (EC) No 1099/2009 of 24 September 2009 on the protection of animals at the time of the killing.

### 4.4. DNA Extraction and 16S rRNA Gene Sequencing Procedure for Cecal Samples

DNA was extracted from 100 mg of cecal contents using the ZymoBIOMICS 96 Magbead DNA Kit from Zymo Research. The sequencing library preparation was performed by following the protocol published by Illumina (document number 15044223, Rev. B). Briefly, the variable regions V3-V4 of the bacterial 16S rRNA gene were targeted by primers designed by Klindworth et al. [38]. In a two-step protocol, the first round of PCR amplified the target and added Illumina sequencing adapters. Nextera XT set A barcodes were added during the second round of PCR. KAPA HiFi DNA polymerase was used for all amplification reactions. Sequencing was performed on an Illumina MiSeq, using the v3 600-cycle reagent kit to produce 2 × 300 bp paired-end reads. A DNA extraction negative control and mock community DNA (ATCC MSA-1002) were sequenced along with the sample libraries for quality control purposes.

### 4.5. Bioinformatics Data Analysis

All of the sequence processing was done within the QIIME2 software environment [39]. First, the primer sequences were trimmed from the reads using Cutadapt [40]. The trimmed reads were denoised with the DADA2 algorithm [41] to produce error-corrected amplicon sequence variants (ASVs). Taxonomic classification of ASVs was performed with VSEARCH [42] against the silva_138_NR99 SSU rRNA database [43].

The alpha and beta diversity calculations were performed on normalized ASV feature tables, from which singletons and sequences representing irrelevant taxa (eukaryotes, chloroplasts, and mitochondria) had been removed. For feature count normalization, the SRS algorithm [44] was used, set to 114,666 features per sample (the lowest denoised and filtered feature count among all samples). The observed feature count (richness), Shannon’s index [45], and Simpson’s index [46] were used as metrics of within-sample (alpha) diversity. Between-sample (beta) diversity was calculated as Bray–Curtis dissimilarity [47] and generalized UniFrac distance [48]. Spearman’s rank correlation tests were performed to assess the relationships between microbiome diversity metrics and feeding supplement dose. A two-sided Mantel test was applied to identify the correlation between beta distances and distances in the feed supplement concentration. Additionally, denoised read count was used as a variable in the same tests in order to assess the sequencing depth as a confounding factor. Associations between feed supplement dose and abundance of microbiome features were analyzed using general linear models as implemented in the MaAsLin2 R package [49].

## 5. Conclusions

Although limited by the small sample size, this pilot study nevertheless demonstrated trends toward dose-dependent desirable changes in the chicken microbiome, resulting from supplementing chicken feed with pine needle extract. Some positive outcomes were observed with increasing doses, such as a reduction in the relative abundance of *Campylobacter.* The effect of pine needle extract on *Campylobacter* abundance and prevalence should be investigated further, especially targeting the species that are pathogenic to the consumers of poultry. In addition, the antimicrobial activity of pine needle extract was also demonstrated against *Enterobacter cloacae*, *Bacillus cereus*, *Salmonella enterica*, and *Acinetobacter baumanii* strains. Consequently, this valuable type of plant biomass can be converted into high value-added nutritional supplements.

## Figures and Tables

**Table 1:** The antimicrobial activities of Baltic pine (*Pinus sylvestris*) needle extract against pathogenic opportunistic microorganisms in liquid medium (+ indicates pathogen growth; − indicates that pathogen growth was not observed).

Pine Extract Volume	Bacterial Strains														
	1	2	3	4	5	6	7	8	9	10	11	12	13	14	15
500 µL	+	+	+	+	+	+	+	+	+	+	+	+	+	+	+
1000 µL	+	+	+	+	+	+	+	+	+	+	-	+	+	+	+
1500 µL	+	+	+	+	+	+	+	+	-	+	-	-	-	+	+
0 µL	+	+	+	+	+	+	+	+	+	+	+	+	+	+	+

The interpretation of results: negative (−)—the pathogens did not grow on the selective culture medium; positive (+)—the pathogens grew on the selective culture medium. 1—*Pseudomonas aeroginosa*; 2—*Enterococcus faecium*; 3—*Enterococcus faecalis*; 4—*Proteus mirabilis*; 5—*E. coli*; 6—*Klebsiella pneumoniae*; 7—*S. aureus*; 8—*S. haemolyticus*; 9—*Enterobacter cloacae*; 10—*Citrobacter freundii*; 11—*Bacillus cereus*; 12—*Salmonella enterica*; 13—*Acinetobacter baumanii*; 14—*Aeromonas hydrophila*; 15—*Pasteurella multocida*.

**Table 2:** Diameters of inhibition zones (mm) of the pine needle extract and *L. plantarum* strain against bacterial strains.

Samples	Diameter of the Inhibition Zone, mm														
	Bacterial Strains														
	1	2	3	4	5	6	7	8	9	10	11	12	13	14	15
0.5 mg/mL	-	-	-	-	-	-	-	-	-	-	-	-	-	-	-
1.0 mg/mL	-	-	-	-	-	-	-	-	-	-	-	-	-	-	-
1.5 mg/mL	-	-	-	-	-	-	-	-	-	-	12.0	-	-	-	-
*L. plantarum*	13.0	10.0	10.0	12.0	9.0	-	-	-	11.0	-	10.0	9.0	11.0	12.0	13.0

1—Pseudomonas aeroginosa; 2—Enterococcus faecium; 3—Enterococcus faecalis; 4—Proteus mirabilis; 5—E. coli; 6—Klebsiella pneumoniae; 7—S. aureus; 8- S. haemolyticus; 9—Enterobacter cloacae; 10—Citrobacter freundii; 11—Bacillus cereus; 12—Salmonella enterica; 13—Acinetobacter baumanii; 14—Aeromonas hydrophila; 15—Pasteurella multocida; (-) no growth inhibition.

**Table 3:** Differentially abundant taxa in the cecal microbiota from birds fed a pine needle extract-supplemented feed.

Phylum	Class	Order	Family	Genus	Coefficient from Linear Model	*p* Value	Q Value
*Bacteroidota*	*Bacteroidia*	*Bacteroidales*	*Bacteroidaceae*	*Bacteroides*	0.0543	0.0126	0.1428
*Bacteroidota*	*Bacteroidia*	*Bacteroidales*	*Barnesiellaceae*		−0.5771	0.0051	0.0956
*Bacteroidota*	*Bacteroidia*	*Bacteroidales*	*Marinifilaceae*	*Odoribacter*	0.2604	0.0010	0.0520
*Bacteroidota*	*Bacteroidia*	*Bacteroidales*	*Prevotellaceae*		0.5776	0.0115	0.1428
*Bacteroidota*	*Bacteroidia*	*Bacteroidales*	*Rikenellaceae*	*Alistipes*	−0.1958	0.0251	0.1963
*Bacteroidota*	*Bacteroidia*	*Bacteroidales*	*Rikenellaceae*	*Rikenellaceae* *RC9 gut group*	−0.5106	0.0273	0.1963
*Campilobacterota*	*Campylobacteria*	*Campylobacterales*	*Campylobacteraceae*	*Campylobacter*	−0.6528	0.0040	0.0956
*Deferribacterota*	*Deferribactere*	*Deferribacterales*	*Deferribacteraceae*	*Mucispirillum*	0.2749	0.0013	0.0520
*Euryarchaeota*	*Methanobacteria*	*Methanobacteriales*	*Methanobacteriaceae*	*Methanobrevibacter*	−0.1979	0.0261	0.1963
*Firmicutes*	*Clostridia*	*Lachnospirales*	*Lachnospiraceae*	*Eubacterium hallii* *group*	−0.1817	0.0060	0.0956
*Synergistota*	*Synergistia*	*Synergistales*	*Synergistaceae*	*Cloacibacillus*	−0.2144	0.0171	0.1685

**Table 4:** Preparation of supplemented feed.

	Week 1	Week 2	Week 3	Week 4
The average body weight per bird	200 g	270 g	340 g	410 g
The daily intake of feed per bird	24 g	29 g	34 g	38 g
The amount of feed for 20 birds for 7 days	3360 g	4060 g	4760 g	5320 g
Group A (non-supplement diet)				
% of supplement in feed	0	0	0	0
Group B (30 mg extract per kg b.w.)				
Amount of daily intake of extract per bird	6.0 mg	8.1 mg	10.2 mg	12.3 mg
Amount of extract for 20 chickens for 7 days	840 mg	1134 mg	1428 mg	1722 mg
% of supplement in feed	0.025	0.027	0.030	0.032
Group C (60 mg extract per kg b.w.)				
Amount of daily intake of extract per bird	12.0 mg	16.2 mg	20.4 mg	24.6 mg
Amount of extract for 20 chickens for 7 days	1680 mg	2268 mg	2856 mg	3444 mg
% of supplement in feed	0.050	0.054	0.060	0.064

## Data Availability

The code and detailed software versions used in the sequence processing and analysis are documented and available at the GitHub repository https://github.com/jkibilds/chicken-feed-microbiome-2021 (accessed on 7 November 2022). All DNA sequencing reads from this study have been deposited at the European Nucleotide Archive under accession number PRJEB46042.
